# A Novel Robust Trilateration Method Applied to Ultra-Wide Bandwidth Location Systems

**DOI:** 10.3390/s17040795

**Published:** 2017-04-07

**Authors:** Jiahong Li, Xianghu Yue, Jie Chen, Fang Deng

**Affiliations:** Key Laboratory of Intelligent Control and Decision of Complex Systems, School of Automation, Beijing Institute of Technology, Beijing 100081, China; lijiahong.bit@gmail.com (J.L.); yuexianghubit@163.com (X.Y.)

**Keywords:** trilateration, intersection determination, confidence level

## Abstract

Due to the non-line-of-sight (NLOS) and multipath fading channel (MPF) of the wireless networks, the non-existence of the intersection point often occurs in the range-based localization methods, e.g., the centroid-based trilateration method. To alleviate the problem, a confidence-based intersection method which expands the range of the circle within a certain confidence interval is proposed. In the method, the confidence interval is estimated based on the Cramér–Rao lower bound of the time of flight (TOF) measurement. Furthermore, an intersection determination method is proposed to select the intersection point with higher confidence level. The simulation and experimental results show the superiority of the proposed method in localization accuracy and robustness to noise compared to the conventional trilateration method, e.g., the centroid-based and least squares based trilateration methods.

## 1. Introduction

Nowadays, with the development of the wireless communication and electrical technology, low-cost, small-size and multi-functional sensor nodes have been largely used in the miliary, healthcare and domestic area [1]. As the basis of the Internet of Things (IOT), the sensor/agent localization is a fundamental task in many applications, e.g., mobile wireless device information sharing, path searching across the in-vehicle networks (IVN), indoor localization and surveillance systems [2,3,4].

Sensor localization has attracted tremendous interest of researchers concentrating on the following three topics. The first topic is how to increase the accuracy of the localization algorithm based on the communication of sensors. One effective approach is to recompute the distance at each sensor by exchanging the computed distance values with the neighbors, as is formulated as a neighbor-based collaborative localization method in [2,5]. The second topic is how to combine the benefits of the several existing localization algorithms. For example, the bounding box method proposed in [6] can obtain higher accuracy with low computation complexity and high coverage speed but is susceptible to the outliers or noise. Therefore, by combining the bounding box method with an outliers-robust trilateration method, both the accuracy and the robustness can be satisfied. The third topic is how to improve or extend the existing algorithms into various environments. One effective approach is to use multiple measurement methods—e.g., [7] proposed an indoor localization method which uses receiver signal strength indicator (RSSI) range and Zigbee communication protocols. In conclusion, the development of the localization algorithms in WSNs (wireless sensor networks) focus on the the algorithm design in complex environments and how to reduce the localization errors.

The ultra-wideband (UWB) technique is a commonly-used technique in the field of wireless sensor network localization. The signal is decomposed in time scale instead of the frequency or band scale, preventing the insurmountable technical barriers of the infinite spectral segment. UWB radios have relative bandwidth larger than 20% or absolute bandwidth of more than 5000 MHz. The operating frequency of UWB is 3.1–10.6 GHz and can produce the narrow pulse directly to activate the antenna. It has very good characteristics such as low power, strong anti-interface capacity, insensitivity to channel fading, good elusiveness, high penetrability and high positioning accuracy [8]. Therefore, the UWB technique is well suited for high accuracy ranging and localization [9].

According to whether the range is measured or not, the localization mechanisms are divided into range-based methods and range-free methods. Range-free schemes, e.g., centroid [10], distance vector-hop (DV)-Hop [11], and approximate point-in-triangulation (APIT) [12], compute the location by exchanging information based on the connectivity of the networks. This scheme is easily implemented; however, it cannot obtain high localization accuracy. Range-based schemes, e.g., the trilateration, triangulation and maximum likelihood (ML) estimation, estimate the location by measuring the point-to-point distance or the angle and have better accuracy [13].

The ranging techniques for range-based methods include time of arrival (TOA), time difference of arrival (TDOA), time of flight (TOF) and arrival of angle (AOA) [14,15]. Among all the techniques, the TOF technique is commonly used in practice for its advantages, e.g., no need for accurate time synchronization, easy implementation and low consumed resources. It computes the range by calculating the time difference between the sender and receiver. For example, in [16], four ultrasonic TOF measurements and navigation position are used to compute the vehicle position based on the extended Kalman filter (EKF). In [17], a new hardware system is established to achieve the attitude tracking and path location for indoor autonomous robots.

According to [13], through the introduction of graph theory, the initial range-based location problem was transformed into a graph problem, which was proven to be an non-deterministic polynomial (NP) complete problem. Therefore, it is necessary to use the numerical methods, e.g., the trilateration method. Trilateration is the process that estimates the location of the target by measuring the distances of itself from three anchor nodes whose positions are already known. The anchors are required to install omnidirectional antenna and compute the distances by measuring the RSSI or other values. Then, the position of the target is estimated based on solving the intersection point of the three circles with the ranges being the radius and the anchor’s position being the center. However, in practice, there are four reasons that will influence the accuracy of the trilateration method, as are discussed below.

1. Uncertainty: Due to the non-line-of-sight (NLOS) and multipath fading channel (MPF) problem, the uncertainty often occurs in the distance measurement procedure, which leads to the phenomenon that there are two intersection points of the anchor circles, and even no points.
2. Heterogeneity: In most cases, the method requires three or more anchor nodes for broader coverage. Therefore, the inconsistency of the computation results may occur.
3. Ambiguity: Flip ambiguity is one kind of ambiguity when a kind of mirror is created to reflect positions [18,19]. It often occurs in noisy channel of distances measurement or sparsely connected networks.
4. Error Propagation:In each iteration step, the error is induced, stored and accumulated, which may deteriorate the location method [20,21,22].

To solve the four problems, Reference [23] proposed a combination of the trilateration with a new geometric method for mobile robot localization. It established a series of geometric formulas including the Carley–Menger determinant to reduce a variety of error inferences, e.g., the multipath error and round-off error. Reference [24] proposed a confidence-based trilateration method which computes the location by selecting the sensors with the confidence level from high to low. At the initial time, the confidence level of each sensor is set according to the conditions, e.g., signal to noise ratio (SNR), the communication range, etc. In the iteration step of the implementation of localization, the confidence level is altered to reduce the localization error. This method can reduce the uncertainty and ambiguity problem efficiently but will increase the computational complexity of WSNs. Reference [25] studied the relationship between network localization and rigidity properties of ground truth graph, and proposed an improved trilateration for wheeled robot localization problem. The diffusion method is used to identify whether the the global grid is localizable or not. This method inherits the simplicity and efficiency of trilateration, and improves the ambiguity by identifying more localizable nodes. Reference [26] proposed the random sample consensus(RANSAC)-based trilateration method to robustly estimate an initial pose graph, which models the locations of sensor platforms. However, it lacks the consideration of two intersection points and non-existence of the intersection points, thus it cannot solve the uncertainty problem.

However, for an indoor environment with obstructions, the above improved methods do not consider the errors, which gives rise to the non-existence of the intersection point, so the narrow usage becomes its shortcoming. For UWB, the errors are composed of systematic errors and random errors. The systematic error stems from the NLOS propagation problem [27,28,29,30], which often occurs in the practical applications of WSN localization with multipath channels. The NLOS is caused by the measurement error induced by the in-network sensor failure. The conventional approach to the NLOS problem is to regard the NLOS error as the additive white Gaussian noise (AWGN) and reduce the interface by the errors based on the variance detection of least squares method (LS). However, it may cause the curse of dimensionality problem when calculating the pseudu-inverse matrix of LS estimate. This paper proposed a novel robust trilateration method to alleviate the problem. In the method, the interface is also modeled as AWGN, and a proper confidence interval is established to solve the non-existence of intersection points. A new intersection determination method is also proposed to reduce the errors induced by uncertainty and ambiguity.

The remainder of this paper is organized as follows. In Section 2, the problem of outlier-robust trilateration is formulated and prior works are illustrated. An improved confidence-based trilateration method with intersection determination criterion is developed in Section 3. The simulation and experimental results show the efficiency of the proposed method in Section 4. Conclusions are demonstrated in Section 6.

## 2. Problem Formulation

Consider the traditional trilateration method with two following assumptions:

**Assumption** **1.** The transmission channel is modeled as an AGWN channel, so the residuals between the measurement of range and the real value satisfies the Gaussian distribution.

**Assumption** **2.** The ranging error induced by the coordination faults (e.g., the hardware and rounding calculation errors) is bounded, and the upper bound is limited to satisfy the Gaussian property of the distance.

Given the two assumptions, the traditional trilateration is given as follows.

Consider a sensor network consisting of *N* anchor nodes and a target deployed over a geographic region. The coordinates of the anchor nodes are known, denoted as $\{\left(x_{i},y_{i}\right)\},i=1,2,\ldots ,N$. $\left(x_{t},y_{t}\right)$ denotes the coordinate of the target node. The distance between each anchor *i* and the target can be calculated by the time of flight (TOF) estimate, denoted as $d_{i}$. Then, the following equations are satisfied:

(1) $${\left(x_{i}-x_{t}\right)}^{2}+{\left(y_{i}-y_{t}\right)}^{2}=d_{i}^{2}\;,i-1,2,\ldots ,N.$$

The intersection point can be derived by solving the simultaneous equations of two nodes *i* and *j* as

(2) $$\left\{\begin{array}{cc} {\left(x_{i}-x_{t}\right)}^{2}+{\left(y_{i}-y_{t}\right)}^{2} & =d_{i}^{2},\\ {\left(x_{j}-x_{t}\right)}^{2}+{\left(y_{j}-y_{t}\right)}^{2} & =d_{j}^{2}, \end{array}\right.$$

where the solutions to the binary quadratic simultaneous equations denoted as $\left(x_{ij},y_{ij}\right)$ and $\left(x_{ij}^{\prime },y_{ij}^{\prime }\right)$ are derived as

(3) $$\left\{\begin{array}{c} x_{ij},x_{ij}^{\prime }=\frac{ab\pm \sqrt{a^{2}b^{2}-\left(1+a^{2}\right)\left(b^{2}-d_{i}^{2}\right)}}{1+a^{2}},\\ y_{ij},y_{ij}^{\prime }=\frac{b\pm a\sqrt{a^{2}b^{2}-\left(1+a^{2}\right)\left(b^{2}-d_{i}^{2}\right)}}{1+a^{2}},\; \end{array}\right.$$

where

$$a=\frac{x_{j}-x_{i}}{y_{j}-y_{i}}$$

$$b=\frac{{\left(x_{j}-x_{i}\right)}^{2}+{\left(y_{j}-y_{i}\right)}^{2}-d_{j}^{2}-d_{i}^{2}}{2\left(y_{j}-y_{i}\right)}.$$

The sufficient condition of the solution is to satisfy the following intersection judgement formula:

(4) $$\left\{\begin{array}{cc} {\left(d_{i}+d_{j}\right)}^{2}-{\left(x_{i}-x_{j}\right)}^{2}-{\left(y_{i}-y_{j}\right)}^{2} & >0,\\ {\left(d_{i}-d_{j}\right)}^{2}-{\left(x_{i}-x_{j}\right)}^{2}-{\left(y_{i}-y_{j}\right)}^{2} & <0. \end{array}\right.$$

**Figure 1:**
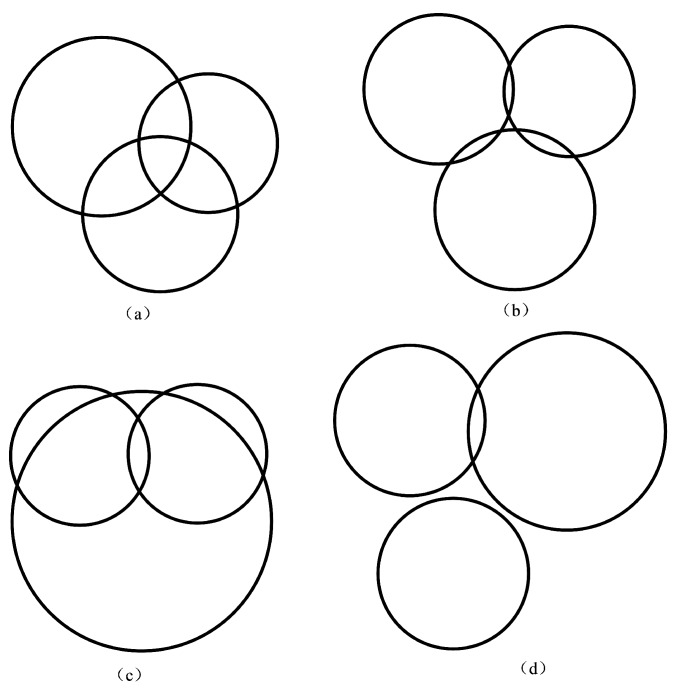
The diagrams of all possibilities of intersections of anchor circles influenced by noise. (**a**) diagram of two intersection points, one of which is outside and one of which is inside of the third circle; (**b**) diagram of two intersection points, both of which are outside of the third circle; (**c**) diagram of two intersection points, both of which are inside of the third circle; and (**d**) diagram of no intersection points.

Ideally, all three of the circles intersect at only one point; however, in practice, the existence of the noises will lead to two intersection points, or even no intersection point, as shown in Figure 1.

As is shown from the figure, Figure 1a–c refer to the two intersections of anchor circles, where the intersection determination criterion is necessary to determine the “best” intersection. In addition, Figure 1d refers to no intersection points, where the distance compensation can be applied to alleviate the problem.

### 2.1. Intersection Determination

Traditional trilateration methods [23,24] adopt the following intersection determination criterion to determine the intersection point that is close to the center of the third circle:

(5) $${\left(x_{ij}-x_{k}\right)}^{2}+{\left(y_{ij}-y_{k}\right)}^{2}<{\left(x_{ij}^{{}^{\prime }}-x_{k}\right)}^{2}+{\left(y_{ij}^{{}^{\prime }}-y_{k}\right)}^{2},$$

where $\left(x_{k},y_{k}\right)$ is the center of the third anchor circle *k* when selecting the “best” intersection point of anchor circle *i* and *j*. If the Equation (5) is satisfied, then select $\left(x_{ij},y_{ij}\right)$ as the “best” intersection point, denoted as $\left(x_{ij}^{☆},y_{ij}^{☆}\right)$. Otherwise, select $\left(x_{ij}^{\prime },y_{ij}^{\prime }\right)$ as $\left(x_{ij}^{☆},y_{ij}^{☆}\right)$.

**Figure 2:**
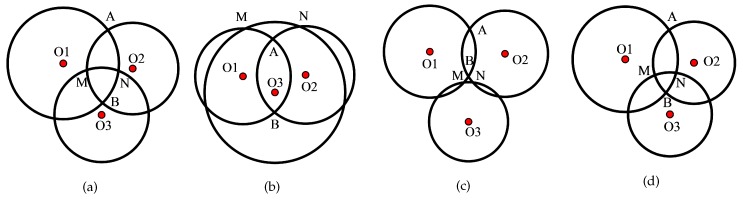
Diagrams of intersection determination schemes.

However, this method cannot be extended to all the possibilities of intersection, as shown in Figure 2.

As is indicated from the figure, the “best” intersection points based on Equation (5) are *B*, *B*, *B* and *B* for Figure 2a–d. However, according to hypotheses 1 and 2, the ranging measurement follows the Gaussian distribution with the mean as the actual range and low variance. Thus, the actual position of the target is more likely to be close to the anchor circle. Specifically, for Figure 2a,b, the real position of the target falls into the triangle region ▵BMN with a higher possibility than the triangle region ▵AMN, and so the real position is *A* and *A* with a high possibility for Figure 2a,b. Therefore, the traditional intersection determination method is sensitive to the noises. To increase the robustness, an improved intersection determination method is proposed in this paper, as shown in Section 3.

### 2.2. Distance Compensation

**Figure 3:**
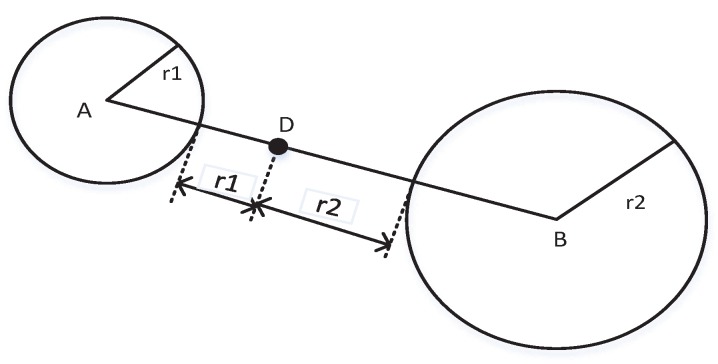
Diagrams of weighted trilateration method.

The distance compensation methods are often applied to solve the non existence problem of the intersection points. It compensates for the distance by extending the radius of the anchor circle when detecting no intersection points of the anchor circles. Reference [31] proposed a weighted trilateration method to obtain the point of the intersection based on the AWGN channel. It calculates the expanded distance as the weighted distance along the line crossing the centers whose weight is computed as the ratio of the radii of circles, as is shown in Figure 3.

However, the method is only suitable for signal-strength based positioning technique, e.g., RSSI, and not suitable for the time-based approach, e.g., TOA and TOF. Furthermore, the presence of large ranging errors induced by NLOS or MPF can also complicate the AWGN channel. These errors make the tails of the distribution of TOF estimates heavier than Gaussian, resulting in the impracticality of the above method in many applications. Therefore, new localization systems should be designed to be robust to these large errors. This paper proposed a novel confidence-based trilateration method based on the Cramér–Rao lower bound (CRLB) of TOF estimate with NLOS propagation in the MPF channel, as shown in Section 3.

## 3. Improved Confidence-Based Trilateration Method

We first give the CRLB of the time of flight (TOF) estimate with NLOS propagation in the MPF channel based on the discussion in [9].

### 3.1. CRLB of TOF Estimation with NLOS Propagation in the MPF Channel

As a time-based positioning technique, the TOF relies on measurements of travel times of signals between nodes. Unlike other time-based techniques, e.g., TOA and TDOA, TOF measures twice the travel times of signals between nodes by letting the receiver send back the signals immediately after it receives them. Therefore, it does not require time synchronization of the nodes. Since the communication channel is modeled as AWGN according to the Assumption 1, it can be shown that the distance estimate $\hat{d}$ satisfies the following inequality:

(6) $$var\left(\hat{d}\right)\geq \frac{c^{2}}{8\pi^{2}SNR\beta^{2}}≜J_{TOF},$$

where *c* is the speed of light, SNR is the signal-to-noise ratio, and β is the effective signal bandwidth defined by

(7) $$\beta ≜{\left[\int_{-\infty }^{\infty }f^{2}{\left|S\left(f\right)\right|}^{2}df/\int_{-\infty }^{\infty }{\left|S\left(f\right)\right|}^{2}df\right]}^{\frac{1}{2}},$$

with S(f) being the Fourier transform of the transmitted signal. As is indicated from the above equations, the CRLB of TOF estimate is only determined by the emission power and the bandwidth of the transmitted signal.

In a UWB positioning system, when the direct LOS between two nodes is blocked, only reflections of the UWB pulse from scatters reach the receiving node. Therefore, the delay of the first arriving pulse does not represent the true TOF. Since the pulse travels an extra distance, a position bias called the NLOS error is present in the measured time delay. Suppose that the probability density function (pdf) of the NLOS delays *l*, $p_{l}\left(l\right)$ is known a priori. According to the CRLB and the Fisher information matrix (FIM), the CRLB of the extra distance induced by the NLOS delay $p_{l}\left(l\right)$ is

(8) $$J_{NLOS}=E\left[\frac{\partial }{\partial l}\ln p_{l}\left(l\right){\left(\frac{\partial }{\partial l}\ln p_{l}\left(l\right)\right)}^{T}\right].$$

A receiver signal with the multipath components is expressed as

(9) $$r\left(t\right)=\sum_{i=1}^{N_{b}}A_{i}s\left(t-\tau_{i}\right)+n\left(t\right),$$

where $A_{i}$ is the amplitude. $\tau_{i}$ is the delay and can be computed as

(10) $$\tau_{i}=\frac{1}{c}\left[\sqrt{{\left(x_{i}-x_{t}\right)}^{2}+{\left(y_{i}-y_{t}\right)}^{2}}\right].$$

The noises n(t) are independent white Gaussian processes with spectral density $\frac{N_{0}}{2}$. Then, the pdf of the received signal r(t) that is conditioned on τ is

(11) $$f_{\tau }\left(r\right)\propto \exp\left\{-\frac{1}{N_{0}}\int_{-\infty }^{\infty }{\left|r\left(t\right)-\sum_{i=1}^{N}A_{i}s\left(t-\tau_{i}\right)\right|}^{2}dt\right\}.$$

The CRLB of the receiver signal with multipath channel is calculated in [32] as:

(12) $$J_{MPF}={\left[\Psi \right]}_{ij},$$

where

(13) $$\begin{array}{cc} \Psi_{ij} & ≜E\left[\frac{\partial }{\partial \tau_{i}}\ln f_{\tau }\left(r\right){\left(\frac{\partial }{\partial \tau_{j}}\ln p_{l}\left(l\right)\right)}^{T}\right]\\ & =\left\{\begin{array}{c} -\frac{2}{N_{0}}R\left[A_{i}A_{j}C\left(\tau_{j}-\tau_{i}\right)\right]\;i\neq j\\ 8\pi^{2}\beta^{2}SNR_{i}\;i==j, \end{array}\right. \end{array}$$

where

(14) $$C_{s}\left(u\right)=\int_{-\infty }^{\infty }s\left(t\right)s^{☆}\left(t-u\right)dt,-\infty <u<\infty$$

is the autocorrelation function of the signal s(t).

In conclusion, the CRLB of the TOF estimate with an NLOS environment in a MPF channel is calculated as

(15) $$J=J_{TOF}+J_{NLOS}+J_{MPF}.$$

### 3.2. New Intersection Determination Principle

As is discussed in the Section 2.1, selecting the intersection points that are close to the center of the third anchor circle is not available. Aiming at the situation, we proposed a new intersection determination principle, as shown below.

**Measurement** **Principle.** For three anchor circles with the centers and radii denoted as $\left(x_{i},y_{i}\right),\left(x_{j},y_{j}\right),\left(x_{k},y_{k}\right)$ and $d_{i},d_{j},d_{k}$, given the intersection points $\left(x_{ij},y_{ij}\right)$ and $\left(x_{ij}^{{}^{\prime }},y_{ij}^{{}^{\prime }}\right)$ of the anchor circle i and j, the optimal intersection point $\left(x_{ij}^{☆},y_{ij}^{☆}\right)$ to calculate the position is selected as

(16) $${\left(d_{k}-\sqrt{{\left(x_{ij}-x_{k}\right)}^{2}+{\left(y_{ij}-y_{k}\right)}^{2}}\right)}^{2}<{\left(d_{k}-\sqrt{{\left(x_{ij}^{{}^{\prime }}-x_{k}\right)}^{2}+{\left(y_{ij}^{{}^{\prime }}-y_{k}\right)}^{2}}\right)}^{2}.$$

*If the inequality condition in the Equation (16) holds, then select $\left(x_{ij},y_{ij}\right)$ as $\left(x_{ij}^{☆},y_{ij}^{☆}\right)$. Otherwise, select $\left(x_{ij}^{{}^{\prime }},y_{ij}^{{}^{\prime }}\right)$ as $\left(x_{ij}^{☆},y_{ij}^{☆}\right)$.*

**Proof** **of** **Measurement** **Principle.** According to the two Assumptions 1–2 and the discussion of the CRLB of the TOF estimate with an NLOS environment in the MPF channel above, the ranging measurements of the sensors ${\hat{d}}_{o},o=i,j,k$ follows the Gaussian distribution with the mean as the true range $d_{o}$ and variance $\sigma_{d_{o}}^{2}$, i.e., ${\hat{d}}_{o}∼N\left(d_{o},\sigma_{d_{o}}^{2}\right),o=i,j,k$. Therefore, the measured range ${\hat{d}}_{o},o=i,j,k$ satisfies the following equation

(17) $$P(-z_{\frac{\alpha }{2}}\leq \frac{|{\hat{d}}_{o}-d_{o}|}{\sigma_{d_{o}}}\leq z_{\frac{\alpha }{2}})=1-\alpha ,$$

where α denotes the confidence coefficient, and is often set to 0.05. $z_{\frac{\alpha }{2}}$ denotes the α quantile and equals 1.96 when α=0.05. Given the intersection points $\left(x_{ij},y_{ij}\right)$ and $\left(x_{ij}^{\prime },y_{ij}^{\prime }\right)$, it is obvious that the one which is close to the circumference of the third anchor circle *k* is more likely to be the true position of the target. Specifically, the intersection points are determined correctly as the “best” intersection points for the diagrams in Figure 2 based on the Equation (16).  ☐

### 3.3. Confidence-Based Distance Compensation

It is known that the range *d* under an NLOS environment in the MPF channel follows the Student-*t* distribution with CRLB computed as the form in Equation (15). Then, the measurement of the distance locates in the confidence interval with a certain confidence level α. δd is denoted as the half of the confidence interval with confidence level set to α=95%, and is calculated as follows:

(18) $$\delta d=1.96J/\sqrt{N}.$$

As is discussed in Section 3, expanding the range measurement of one anchor node is one effective approach. For the non-existence of intersection of the *i*th anchor node and *j*th anchor node, the radius of the anchor circle $d_{s},s=i,j$ is expanded by the new distance $d_{s}^{☆}=d+\delta d$.

### 3.4. Positioning Method

Then, the procedure of the proposed trilateration method is developed as follows.

1. For *q*th selection, select three anchor nodes from *N* nodes which positions are known, denoted as i,j,k. Then, the positions satisfy the Equation (1).
2. Simultaneous the two equations of the *i*th anchor and *j*th anchor. Applying the intersection judgement formula in (4), if the solutions to the equations exist, then go to step 4; otherwise, go to step 3.
3. The range *d* is compensated by the new distance $d^{☆}=d+\delta d$, where δd is calculated in the Equation (18). Go to step 2.
4. The coordinates of the intersections are shown as the form in the Equation (3). Based on the proposed intersection determination criterion in the Equation (16), the optimal intersection point $\left(x_{ij}^{☆},y_{ij}^{☆}\right)$ is determined.
5. Another two optimal intersection points $\left(x_{ik}^{☆},y_{ik}^{☆}\right)$ and $\left(x_{jk}^{☆},y_{jk}^{☆}\right)$ are determined following the steps 1–4. Based on the centroid-based trilateration method, the coordination of the target of *q*th combination can be computed as:
  (19) $$\begin{array}{cc} {\hat{x}}_{t}^{q} & =\frac{1}{3}\left(x_{ij}^{☆}+x_{ik}^{☆}+x_{jk}^{☆}\right),\\ {\hat{y}}_{t}^{q} & =\frac{1}{3}\left(y_{ij}^{☆}+y_{ik}^{☆}+y_{jk}^{☆}\right). \end{array}$$
6. By alternating the anchor nodes, then the coordination of the target can be computed following steps 1–5. Then, the estimate of the coordination of the target is computed as:
  (20) $$\begin{array}{cc} {\hat{x}}_{t}^{☆} & =\frac{1}{C_{N}^{3}}\sum_{q=1}^{C_{N}^{3}}{\hat{x}}_{t}^{q},\\ {\hat{y}}_{t}^{☆} & =\frac{1}{C_{N}^{3}}\sum_{q=1}^{C_{N}^{3}}{\hat{y}}_{t}^{q}. \end{array}$$

## 4. Results

### 4.1. Experiments

**Figure 4:**
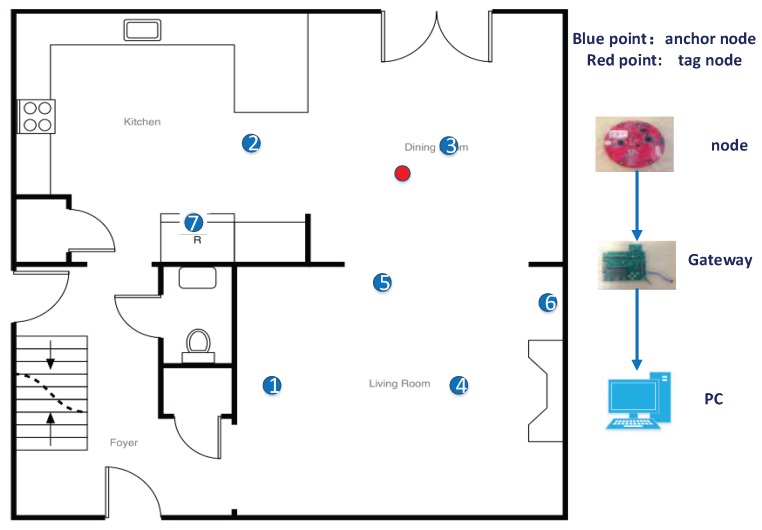
The diagram of the sensor deployment.

In the experiments, there are seven anchor nodes and one tag node (target) initially placed in a 25 m × 25 m square room, as shown in Figure 4. The hardware platforms of the anchor nodes, the tag node and gateway to the upper computer are shown on the right side of the figure. The DecaWave DWM1000 module, Dublin, Ireland is selected as the UWB ranging device that is installed on all the anchor nodes and the tag node. The TI SmartRF CC2538EM module, Dallas, TX, USA is selected as the communication device between all of the devices. Since the two-way ranging does not require accurate time synchronization, the time of flight method is applied to calculate the range by measuring elapsed time for a transmission between a tag and an anchor node based on the estimated propagation speed of an UWB signal through a medium.

The coordinates of the anchor nodes are set to (0,0), (0,14.9), (7.76,14.9), (7.76,0), (5,5), (12,6), (5,10), and (5.6,12.8), and the unit is meters. It should be noted that there is an obstruction between the seventh anchor node and the tag node. Therefore, the seventh anchor circle cannot intersect with other anchor circles due to the NLOS environment and the multipath fading channel.

### 4.2. Accuracy Performance

To show the efficiency of the proposed trilateration method in terms of the location accuracy, the root mean square error (RMSE) of the location is introduced as follows:

(21) $$e_{RMSE}=\frac{1}{C_{N}^{3}}\sqrt{\left[\sum_{i=1}^{C_{N}^{3}}{\left({\hat{x}}_{i}^{t}-x^{t}\right)}^{2}+{\left({\hat{y}}_{i}^{t}-y^{t}\right)}^{2}\right],}$$

where ${\hat{x}}_{i}^{t}$ is the estimate of the position of the *i*th selection scheme. *N* is the number of the selected sensors.

For comparison, we also ran the least squares method(LS)-based trilateration method on the same devices. The LS-based trilateration method is to compute the location estimate of the target ${\hat{X}}_{t}=\left[{\hat{x}}_{t},{\hat{y}}_{t}\right]$ as follows:

(22) $${\hat{X}}_{t}={\left(A^{T}A\right)}^{-1}A^{T}b,$$

where

(23) $$A=\left[\begin{array}{cc} 2(x_{1}-x_{N}) & 2(y_{1}-y_{N})\\ 2(x_{1}-x_{N}) & 2(y_{2}-y_{N})\\ \vdots & \vdots \\ 2(x_{N-1}-x_{N}) & 2(y_{1}-y_{N}) \end{array}\right],$$

(24) $$b=\left[\begin{array}{c} x_{1}^{2}+y_{1}^{2}-x_{N}^{2}-y_{N}^{2}-d_{1}^{2}+d_{N}^{2}\\ x_{2}^{2}+y_{2}^{2}-x_{N}^{2}-y_{N}^{2}-d_{2}^{2}+d_{N}^{2}\\ \vdots \\ x_{N-1}^{2}+y_{N-1}^{2}-x_{N}^{2}-y_{N}^{2}-d_{N-1}^{2}+d_{N}^{2} \end{array}\right].$$

**Figure 5:**
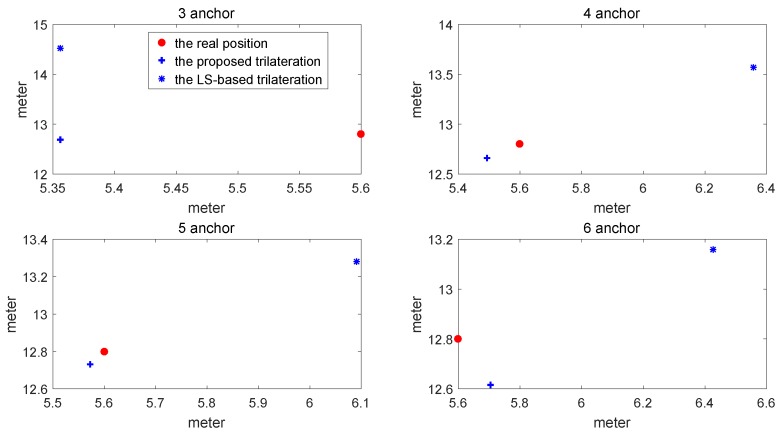
Comparison of the estimated locations between the proposed method and the LS-based trilateration method.

**Figure 6:**
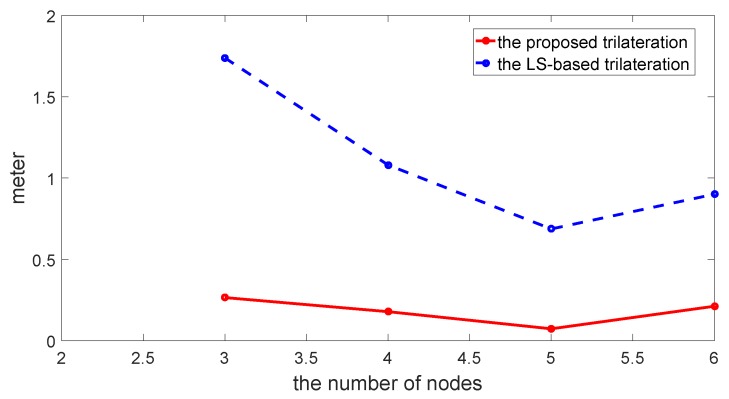
Comparison of RMSE varying with numbers of sensors between the proposed method and the LS-based trilateration method.

The comparison of location estimate between the proposed method and LS-based trilateration method is shown in Figure 5. The comparison of RMSE varying with the number of sensors between the proposed method and the conventional LS-based trilateration method is shown in Figure 6 and Table 1.

As is indicated from the above figures and the table, the RMSE of the proposed method is much lower than that of the LS-based trilateration method. Furthermore, even though the number of sensors is less than four, the RMSE of the proposed method is still less than 0.3. Therefore, the proposed trilateration method is efficient in terms of location accuracy.

### 4.3. Robustness Performance

To show the robustness of the proposed method, we reduce the range of each anchor node by 0.1 m and introduce the seventh anchor node. Thus, it may appear that two anchor circles may not intersect with each other.

**Figure 7:**
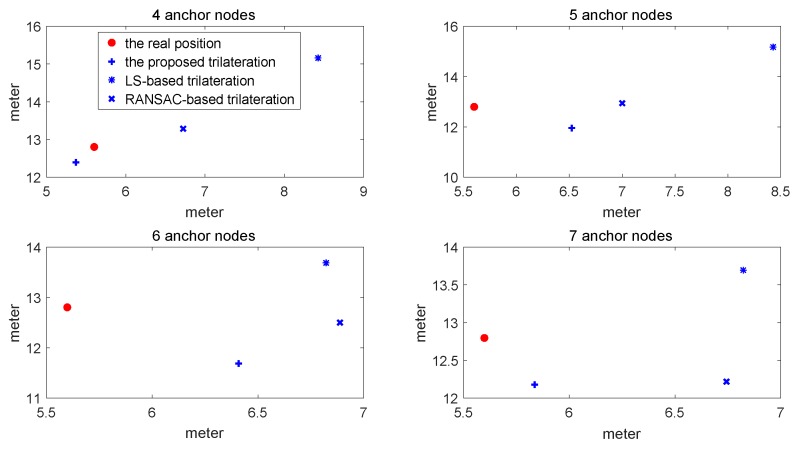
Comparison of the estimated location among the proposed method, the LS-based trilateration method and the RANSAC-based method.

**Figure 8:**
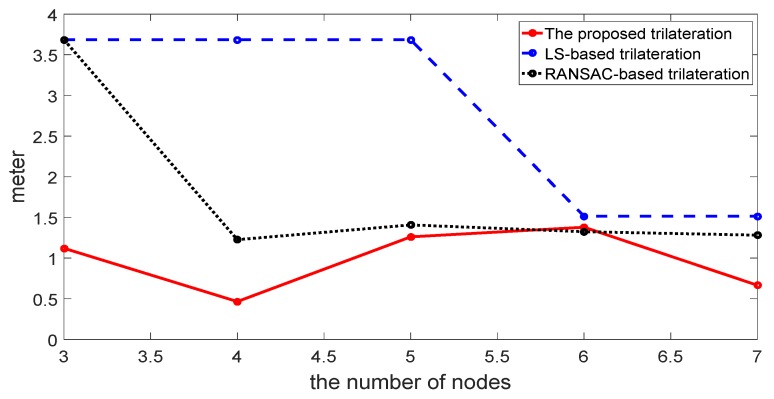
Comparison of RMSE varying with numbers of sensors among the proposed method, the LS-based trilateration method and the RANSAC-based method.

For comparisons, we also ran the LS-based trilateration method with the weighted method shown in [31] and the RANSAC-based robust trilateration method shown in [26]. In the RANSAC-based robust trilateration method, the general trilateration method is used to estimate the target and uses three consistency criterions to evaluate the correctness of the location estimate. The comparisons of the location estimate between these methods are shown in Figure 7. The comparisons of RMSE varying with the numbers of sensors are shown in Figure 8 and Table 2.

As is indicated from the above figures and the table, the RMSE of the proposed method is lower than that of the LS-based trilateration method and RANSAC-based trilateration method. Furthermore, when the seventh anchor node is added to compute the location, the RMSE of the proposed method is reduced whilst that of other methods are not, which shows that its distance is influenced by NLOS and the multipath fading channel is well compensated. Therefore, the proposed trilateration method is efficient in terms of robustness to outliers.

## 5. Discussion

In Figure 5 and Figure 6, the proposed trilateration has better estimated location and less RMSE than the LS-based trilateration varying with numbers of sensors. Thus, when the intersection points exist, the new intersection determination principle can select the intersection points which are close to the center of the third anchor circle and show good characteristics in solving the consistency and ambiguity problem. According to Figure 7 and Figure 8, the proposed positioning method based confidence-based distance compensation could make two circles that have no intersection points intersect again at a certain level of confidence. Therefore, the proposed method has better robustness performance than the RANSAC-based trilateration method and the LS-based trilateration method. In summary, as is indicated from the figures and the tables, the proposed method has better performance in accuracy and robustness than the LS-based method and the RANSAC-based trilateration method.

## 6. Conclusions

This paper proposed a new confidence-based robust trilateration method to solve the NLOS errors and MPF errors. A new intersection determination criterion for all the possibilities of intersection is provided to solve the consistency and ambiguity problem. For the non-existence of intersection points, a confidence-based distance compensation method is proposed to make two circles intersect again. The theoretical analysis is provided to show the efficiency of the method. The experimental results show the superiority of the proposed method compared to LS-based and RANSAC-based trilateration in terms of accuracy and robustness to outliers. Thus, the method can largely reduce the computation burden and improve the communication rate and the survivability of the sensors. 

## Figures and Tables

**Table 1:** Comparison of RMSE between the proposed method and conventional LS-based method (m).

Selection Schemes of the Anchor Nodes	Proposed Method	LS-Based Trilateration
ID: 1, 2, 3	0.2649	1.7385
ID: 1, 2, 3, 4	0.1783	1.0801
ID: 1, 2, 3, 4, 5	0.0729	0.6868
ID: 1, 2, 3, 4, 5, 6	0.2117	0.9004

**Table 2:** Comparison of RMSE among the proposed method, the LS-based trilateration method and the RANSAC-based method (m).

Selection Schemes of the Anchor Nodes	Proposed Method	LS-Based Method	RANSAC-Based Method
ID:1,2,3	1.1200	3.6839	3.6839
ID:1,2,3,4	0.4651	3.6839	1.2258
ID:1,2,3,4,5	1.2605	3.6839	1.4073
ID:1,2,3,4,5,6	1.3790	1.5140	1.3239
ID:1,2,3,4,5,6,7	0.6654	1.5140	1.2814
